# Supplementary material for: Smooth Interpolating Curves with Local Control and Monotone Alternating Curvature
Source: Comput Graph Forum. 2022 Oct 6;41(5):25–38. doi: 10.1111/cgf.14600 (PMC9827861; doi:10.1111/cgf.14600)
Supplement: Supplementary file 1 — Supplement Material [file CGF-41-25-s001.zip › Local-Smooth-Interpolating-MonoCurvature/extern/clothoids/docs/api-cpp/program_listing_file_G2lib.cc.html]

Program Listing for File G2lib.cc — Clothoids v2.0.9

### Navigation

- index
- toc
- Clothoids »
- Program Listing for File G2lib.cc

# Program Listing for File G2lib.cc¶

↰ Return to documentation for file (`G2lib.cc`)

```
/*--------------------------------------------------------------------------*\
 |                                                                          |
 |  Copyright (C) 2017                                                      |
 |                                                                          |
 |         , __                 , __                                        |
 |        /|/  \               /|/  \                                       |
 |         | __/ _   ,_         | __/ _   ,_                                |
 |         |   \|/  /  |  |   | |   \|/  /  |  |   |                        |
 |         |(__/|__/   |_/ \_/|/|(__/|__/   |_/ \_/|/                       |
 |                           /|                   /|                        |
 |                           \|                   \|                        |
 |                                                                          |
 |      Enrico Bertolazzi                                                   |
 |      Dipartimento di Ingegneria Industriale                              |
 |      Universita` degli Studi di Trento                                   |
 |      email: enrico.bertolazzi@unitn.it                                   |
 |                                                                          |
\*--------------------------------------------------------------------------*/

#include "Clothoids.hh"
#include "PolynomialRoots.hh"

#include <algorithm>

#ifdef __clang__
#pragma clang diagnostic ignored "-Wglobal-constructors"
#endif

namespace G2lib {

  using std::numeric_limits;
  using std::fpclassify;
  using std::lower_bound;
  using std::abs;
  using std::sqrt;
  using std::sin;
  using std::cos;
  using std::tan;
  using std::atan;
  using std::asin;
  using std::acos;

  real_type const m_1_sqrt_pi  = 0.564189583547756286948079451561; // 1/sqrt(pi)

  real_type const machepsi     = numeric_limits<real_type>::epsilon();
  real_type const machepsi10   = 10*machepsi;
  real_type const machepsi100  = 100*machepsi;
  real_type const machepsi1000 = 1000*machepsi;
  real_type const sqrtMachepsi = sqrt(machepsi);
  bool            intersect_with_AABBtree = true;

  #ifdef G2LIB_COMPATIBILITY_MODE
  bool use_ISO = true;
  #endif

  char const *CurveType_name[] = {
    "LINE",
    "POLYLINE",
    "CIRCLE",
    "BIARC",
    "BIARC_LIST",
    "CLOTHOID",
    "CLOTHOID_LIST"
  };

  void
  rangeSymm( real_type & ang ) {
    ang = fmod( ang, Utils::m_2pi );
    while ( ang < -Utils::m_pi ) ang += Utils::m_2pi;
    while ( ang >  Utils::m_pi ) ang -= Utils::m_2pi;
  }

  #ifndef DOXYGEN_SHOULD_SKIP_THIS
  static inline real_type power2( real_type a ) { return a*a; }
  static inline real_type power3( real_type a ) { return a*a*a; }
  #endif

  // - - - - - - - - - - - - - - - - - - - - - - - - - - - - - - - - - - - - - -

  /*
  // sin(x)/x
  */
  real_type
  Sinc( real_type x ) {
    if ( abs(x) < 0.02 ) {
      real_type x2 = x*x;
      return 1-(x2/6)*(1-(x2/20)*(1-x2/42));
    } else {
      return sin(x)/x;
    }
  }

  real_type
  Sinc_D( real_type x ) {
    real_type x2 = x*x;
    if ( abs(x) < 0.04 ) return -(x/3)*(1-(x2/10)*(1-(x2/28)*(1-(x2/54))));
    else                 return (cos(x)-sin(x)/x)/x;
  }

  real_type
  Sinc_DD( real_type x ) {
    real_type x2 = x*x;
    if ( abs(x) < 0.02 ) return -1./3.+x2*(0.1-x2*((1.0/168.0)-(x2/6480)));
    else                 return ((2/x2-1)*sin(x)-2*cos(x)/x)/x;
  }

  real_type
  Sinc_DDD( real_type x ) {
    real_type x2 = x*x;
    if ( abs(x) < 0.009 ) return (1.0/5.0+(-1.0/42.0+(1.0/1080.0)*x2)*x2)*x;
    else                  return ((6/x2-1)*cos(x)+(3-6/x2)*sin(x)/x)/x;
  }

  // - - - - - - - - - - - - - - - - - - - - - - - - - - - - - - - - - - - - - -

  /*
  // (1-cos(x))/x
  */
  real_type
  Cosc( real_type x ) {
    if ( abs(x) < 0.04 ) {
      real_type x2 = x*x;
      return (x/2)*(1-(x2/12)*(1-(x2/30)*(1-x2/56)));
    } else {
      return (1-cos(x))/x;
    }
  }

  real_type
  Cosc_D( real_type x ) {
    if ( abs(x) < 0.02 ) {
      real_type x2  = x*x;
      return 0.5*(1-(x2/4)*(1-(x2/18)*(1-(x2/40))));
    } else {
      return (sin(x)+(cos(x)-1)/x)/x;
    }
  }

  real_type
  Cosc_DD( real_type x ) {
    real_type x2  = x*x;
    if ( abs(x) < 0.04 )
      return -(x/4)*(1-(x2/9)*(1-((3.0/80.0)*x2)*(1-((2.0/105.0)*x2))));
    else
      return ((1-2/x2)*cos(x)+(2/x-sin(x))/x)/x;
  }

  real_type
  Cosc_DDD( real_type x ) {
    real_type x2 = x*x;
    if ( abs(x) < 0.02 )
      return -(1-(x2/3)*(1-(x2/16)*(1-(2.0/75.0)*x2)))/4.0;
    else
      return ((6/x2-1)*sin(x)+((6/x2-3)*cos(x)-6/x2)/x)/x;
  }

  // - - - - - - - - - - - - - - - - - - - - - - - - - - - - - - - - - - - - - -

  /*
  // atan(x)/x
  */
  real_type
  Atanc( real_type x ) {
    if ( abs(x) < 0.03 ) {
      real_type x2 = x*x;
      return 1-x2*((1./3.)-x2*((1./5.)-x2*((1./7.)-x2*((1./9.)-(x2/11)))));
    } else {
      return atan(x)/x;
    }
  }

  real_type
  Atanc_D( real_type x ) {
    real_type x2 = x*x;
    if ( abs(x) < 0.03 ) {
      return x*( -(2./3.) + x2*( (4./5.) + x2*( -(6./7.) + x2*( (8./9.) + x2*( -(10./11.) + x2*(12./13.))))));
    } else {
      return (1/(1+x2)-atan(x)/x)/x;
    }
  }

  real_type
  Atanc_DD( real_type x ) {
    real_type x2 = x*x;
    if ( abs(x) < 0.02 ) {
      return -2./3.+ x2*( (12./5.) + (-(30./7.) + x2 * ( (56./9.) + x2*( -(90./11.) + x2 * (132./13.)))));
    } else {
      return (2*atan(x)/x-(4*x2+2)/power2(1+x2))/x2;
    }
  }

  real_type
  Atanc_DDD( real_type x ) {
    real_type x2 = x*x;
    if ( abs(x) < 0.02 ) {
      return x*(24./5.+x2*(-120./7. + x2 * (112./3. + x2 * (-720./11. + x2*(1320./13. - x2*728./5.)))));
    } else {
      return ( ((18*x2+16)*x2+6)/power3(x2+1)-6*atan(x)/x )/(x2*x);
    }
  }

  // - - - - - - - - - - - - - - - - - - - - - - - - - - - - - - - - - - - - - -

  #ifndef DOXYGEN_SHOULD_SKIP_THIS
  static
  real_type
  maxabs3( real_type A, real_type B, real_type C ) {
    real_type res  = abs(A);
    real_type absB = abs(B);
    if ( res < absB ) res = absB;
    real_type absC = abs(C);
    if ( res < absC ) res = absC;
    return res;
  }
  #endif

  int_type
  solveLinearQuadratic(
    real_type   A,
    real_type   B,
    real_type   C,
    real_type   a,
    real_type   b,
    real_type   c,
    real_type * x,
    real_type * y
  ) {
    real_type m1 = maxabs3(A,B,C);
    real_type m2 = maxabs3(a,b,c);
    A /= m1; B /= m1; C /= m1;
    a /= m2; b /= m2; c /= m2;
    real_type Ab   = A * b;
    real_type Ba   = B * a;
    real_type tmp  = A * Ab + B * Ba;
    real_type disc = tmp - (C * C) * a * b;
    real_type ACb  = Ab*C;
    real_type BCa  = Ba*C;
    if ( disc > machepsi100 ) {
      // two solution
      disc = sqrt(disc);
      real_type Bdisc = B*disc;
      real_type Adisc = A*disc;
      x[0] = (ACb-Bdisc)/tmp;
      x[1] = (ACb+Bdisc)/tmp;
      y[0] = (BCa+Adisc)/tmp;
      y[1] = (BCa-Adisc)/tmp;
      return 2;
    } if ( disc > -machepsi100 ) {
      // one solution
      x[0] = ACb/tmp;
      y[0] = BCa/tmp;
      return 1;
    }
    return 0; // no solution
  }

  // - - - - - - - - - - - - - - - - - - - - - - - - - - - - - - - - - - - - - -

  int_type
  solveLinearQuadratic2(
    real_type   A,
    real_type   B,
    real_type   C,
    real_type * x,
    real_type * y
  ) {
    real_type m = maxabs3(A,B,C);
    A /= m; B /= m; C /= m;
    real_type tmp  = A*A + B*B;
    real_type disc = tmp - (C * C);
    real_type AC   = A*C;
    real_type BC   = B*C;
    if ( disc > machepsi100 ) {
      // two solution
      disc = sqrt(disc);
      real_type Bdisc = B*disc;
      real_type Adisc = A*disc;
      x[0] = (AC-Bdisc)/tmp;
      x[1] = (AC+Bdisc)/tmp;
      y[0] = (BC+Adisc)/tmp;
      y[1] = (BC-Adisc)/tmp;
      return 2;
    } if ( disc > -machepsi100 ) {
      // one solution
      x[0] = AC/tmp;
      y[0] = BC/tmp;
      return 1;
    }
    return 0; // no solution
  }

  // - - - - - - - - - - - - - - - - - - - - - - - - - - - - - - - - - - - - - -

  #ifndef DOXYGEN_SHOULD_SKIP_THIS

  static
  int_type
  solveNLsysCircleCircle(
    real_type kA,
    real_type T,
    real_type Tx,
    real_type Ty,
    real_type kB,
    real_type x[2],
    real_type y[2]
  ) {
    real_type Tx2 = Tx*Tx;
    real_type Ty2 = Ty*Ty;
    real_type kB2 = kB*kB;
    real_type kA2 = kA*kA;
    real_type a   = (Tx2+Ty2)*kB2/4+Tx*kA*kB+kA2;
    real_type b   = (Tx*kB+2*kA)*T-Ty2;
    real_type c   = T*T;
    PolynomialRoots::Quadratic qsolve( a, b, c);
    if ( qsolve.complexRoots() ) return 0;
    real_type z[2] = { qsolve.real_root0(), qsolve.real_root1() };
    int_type nint = 0;
    for ( int_type i = 0; i < qsolve.numRoots(); ++i ) {
      real_type tmp = z[i]*(4-kB2*z[i]);
      if ( tmp < 0 ) continue;
      real_type xx = kB*z[i]/2;
      real_type yy = sqrt( tmp )/2;
      tmp = Tx*xx+kA*z[i]+T;
      if ( abs(tmp-Ty*yy) < abs(tmp+Ty*yy) ) yy = -yy;
      x[nint] = xx;
      y[nint] = yy;
      ++nint;
    }
    return nint;
  }

  static
  real_type
  invCoscSinc( real_type k, real_type x, real_type y ) {
    real_type ds, s = y;
    if ( abs(k) > sqrtMachepsi ) s = atan2( y*k, 1-k*x )/k;
    int_type iter = 0;
    do {
      real_type sk = s*k;
      ds = (y-Sinc(sk)*s)*cos(sk)/(1-sin(sk)*k*y);
      s += ds;
    } while ( abs(ds) > machepsi100 && ++iter < 5 );
    return s;
  }

  #endif

  int_type
  intersectCircleCircle(
    real_type   x1,
    real_type   y1,
    real_type   theta1,
    real_type   kappa1,
    real_type   x2,
    real_type   y2,
    real_type   theta2,
    real_type   kappa2,
    real_type * s1,
    real_type * s2
  ) {
    real_type dx    = x2 - x1;
    real_type dy    = y2 - y1;
    real_type L2    = dx*dx+dy*dy;
    real_type L     = sqrt(L2);
    real_type alpha = atan2( dy, dx );
    real_type a1    = alpha-theta1;
    real_type a2    = alpha-theta2;
    real_type t12   = theta1-theta2;
    real_type Sa1   = L*sin(a1);
    real_type Ca1   = L*cos(a1);
    real_type Sa2   = L*sin(a2);
    real_type Ca2   = L*cos(a2);
    real_type S12   = sin(t12);
    real_type C12   = cos(t12);
    real_type T1    = L2*kappa2+2*Sa2;
    real_type T2    = L2*kappa1-2*Sa1;
    real_type xx1[2], yy1[2], xx2[2], yy2[3];
    int_type nsol;
    if ( abs(T1) > abs(T2) ) {
      real_type Tx1 = -2*(Sa1*kappa2+C12);
      real_type Ty1 = -2*(Ca1*kappa2+S12);
      nsol = solveNLsysCircleCircle( kappa2, T1, Tx1, Ty1, kappa1, xx1, yy1 );
      for ( int_type i = 0; i < nsol; ++i ) {
        xx2[i] = C12*xx1[i]+S12*yy1[i]-Sa2;
        yy2[i] = C12*yy1[i]-S12*xx1[i]-Ca2;
      }
    } else {
      real_type Tx2 = 2*(Sa2*kappa1-C12);
      real_type Ty2 = 2*(Ca2*kappa1+S12);
      nsol = solveNLsysCircleCircle( kappa1, T2, Tx2, Ty2, kappa2, xx2, yy2 );
      for ( int_type i = 0; i < nsol; ++i ) {
        xx1[i] = C12*xx2[i]-S12*yy2[i]+Sa1;
        yy1[i] = C12*yy2[i]+S12*xx2[i]+Ca1;
      }
    }
    real_type len1 = Utils::m_2pi/(machepsi+abs(kappa1));
    real_type len2 = Utils::m_2pi/(machepsi+abs(kappa1));
    for ( int_type i = 0; i < nsol; ++i ) {
      real_type ss1 = invCoscSinc( kappa1, xx1[i], yy1[i] );
      real_type ss2 = invCoscSinc( kappa2, xx2[i], yy2[i] );
      while ( ss1 < 0    ) ss1 += len1;
      while ( ss2 < 0    ) ss2 += len2;
      while ( ss1 > len1 ) ss1 -= len1;
      while ( ss2 > len2 ) ss2 -= len2;
      s1[i] = ss1;
      s2[i] = ss2;
    }
    return nsol;
  }

  /*\
   |  __  ____   __  _          _____ _          _
   |  \ \/ /\ \ / / | |_ ___   |_   _| |__   ___| |_ __ _
   |   \  /  \ V /  | __/ _ \    | | | '_ \ / _ \ __/ _` |
   |   /  \   | |   | || (_) |   | | | | | |  __/ || (_| |
   |  /_/\_\  |_|    \__\___/    |_| |_| |_|\___|\__\__,_|
  \*/

  void
  xy_to_guess_angle(
    int_type          npts,
    real_type const * x,
    real_type const * y,
    real_type       * theta,
    real_type       * theta_min,
    real_type       * theta_max,
    real_type       * omega,
    real_type       * len
  ) {
    //
    // Compute guess angles
    //
    int_type ne  = npts-1;
    int_type ne1 = npts-2;

    real_type dx = x[1]-x[0];
    real_type dy = y[1]-y[0];
    omega[0] = atan2(dy,dx);
    len[0]   = hypot(dy,dx);
    for ( int_type j = 1; j < ne; ++j ) {
      dx       = x[j+1]-x[j];
      dy       = y[j+1]-y[j];
      omega[j] = atan2(dy,dx);
      len[j]   = hypot(dy,dx);
      real_type domega = omega[j]-omega[j-1];
      domega  -= round(domega/Utils::m_2pi)*Utils::m_2pi;
      omega[j] = omega[j-1]+domega;
    }

    real_type const dangle = 0.99 * Utils::m_pi;

    theta[0]      = omega[0];
    theta_min[0]  = omega[0] - dangle;
    theta_max[0]  = omega[0] + dangle;

    theta[ne]     = omega[ne1];
    theta_min[ne] = omega[ne1] - dangle;
    theta_max[ne] = omega[ne1] + dangle;

    real_type omega_L = omega[0];
    real_type len_L   = len[0];
    for ( int_type j = 1; j < ne; ++j ) {
      real_type omega_R = omega[j];
      real_type len_R   = len[j];
      theta[j] = ( omega_L/len_L + omega_R / len_R ) / ( 1/len_L + 1/len_R );
      if ( omega_R > omega_L ) {
        theta_min[j] = omega_L;
        theta_max[j] = omega_R;
      } else {
        theta_min[j] = omega_R;
        theta_max[j] = omega_L;
      }
      theta_min[j] -= dangle;
      theta_max[j] += dangle;
      omega_L = omega_R;
      len_L   = len_R;
    }
  }

  /*\
   |   ____        _           ____       ____
   |  / ___|  ___ | |_   _____|___ \__  _|___ \
   |  \___ \ / _ \| \ \ / / _ \ __) \ \/ / __) |
   |   ___) | (_) | |\ V /  __// __/ >  < / __/
   |  |____/ \___/|_| \_/ \___|_____/_/\_\_____|
  \*/

  bool
  Solve2x2::factorize( real_type A[2][2] ) {
    // full pivoting
    real_type Amax = abs(A[0][0]);
    real_type tmp  = abs(A[0][1]);
    int_type ij = 0;
    if ( tmp > Amax ) { ij = 1; Amax = tmp; }
    tmp = abs(A[1][0]);
    if ( tmp > Amax ) { ij = 2; Amax = tmp; }
    tmp = abs(A[1][1]);
    if ( tmp > Amax ) { ij = 3; Amax = tmp; }
    if ( Utils::isZero(Amax) ) return false;
    if ( (ij&0x01) == 0x01 ) { j[0] = 1; j[1] = 0; }
    else                     { j[0] = 0; j[1] = 1; }
    if ( (ij&0x02) == 0x02 ) { i[0] = 1; i[1] = 0; }
    else                     { i[0] = 0; i[1] = 1; }
    // apply factorization
    LU[0][0] = A[i[0]][j[0]];
    LU[0][1] = A[i[0]][j[1]];
    LU[1][0] = A[i[1]][j[0]];
    LU[1][1] = A[i[1]][j[1]];

    LU[1][0] /= LU[0][0];
    LU[1][1] -= LU[1][0]*LU[0][1];
    // check for singularity
    singular = abs( LU[1][1] ) < epsi;
    return true;
  }

  // - - - - - - - - - - - - - - - - - - - - - - - - - - - - - - - - - - - - - -

  bool
  Solve2x2::solve( real_type const b[2], real_type x[2] ) const {
    if ( singular ) {
      // L^+ Pb
      real_type tmp = (b[i[0]] + LU[1][0]*b[i[1]]) /
                      ( (1+power2(LU[1][0]) ) * ( power2(LU[0][0])+power2(LU[0][1]) ) );
      x[j[0]] = tmp*LU[0][0];
      x[j[1]] = tmp*LU[0][1];
      // check consistency
      tmp = (LU[0][0]*x[j[0]]+LU[0][1]*x[j[1]]);
      return hypot( b[i[0]]-tmp, b[i[1]]+tmp*LU[1][0] ) < hypot(b[0],b[1])*epsi;
    } else { // non singular
      // L^(-1) Pb
      x[j[0]] = b[i[0]];
      x[j[1]] = b[i[1]]-LU[1][0]*x[j[0]];
      // U^(-1) x
      x[j[1]] /= LU[1][1];
      x[j[0]]  = (x[j[0]]-LU[0][1]*x[j[1]])/LU[0][0];
      return FP_INFINITE != fpclassify(x[0]) &&
             FP_NAN      != fpclassify(x[0]) &&
             FP_INFINITE != fpclassify(x[1]) &&
             FP_NAN      != fpclassify(x[1]);
    }
  }

  // - - - - - - - - - - - - - - - - - - - - - - - - - - - - - - - - - - - - - -

  int_type
  isCounterClockwise(
    real_type const * P1,
    real_type const * P2,
    real_type const * P3
  ) {
    real_type dx1 = P2[0] - P1[0];
    real_type dy1 = P2[1] - P1[1];
    real_type dx2 = P3[0] - P1[0];
    real_type dy2 = P3[1] - P1[1];
    real_type tol = machepsi10*(hypot(dx1,dy1)*hypot(dx2,dy2));
    real_type det = dx1*dy2 - dy1*dx2;
    if      ( det >  tol ) return  1;
    else if ( det < -tol ) return -1;
    return 0;
  }

  // - - - - - - - - - - - - - - - - - - - - - - - - - - - - - - - - - - - - - -

  real_type
  projectPointOnCircleArc(
    real_type x0,
    real_type y0,
    real_type c0,
    real_type s0,
    real_type k,
    real_type L,
    real_type qx,
    real_type qy
  ) {
    real_type dx  = x0 - qx;
    real_type dy  = y0 - qy;
    real_type a0  = c0 * dy - s0 * dx;
    real_type b0  = s0 * dy + c0 * dx;
    real_type tmp = a0*k;

    if ( 1+2*tmp > 0 ) {

      tmp = b0/(1+tmp);
      tmp *= -Atanc(tmp*k); // lunghezza

      if ( tmp < 0 ) {
        real_type absk = abs(k);
        // if 2*pi*R + tmp <= L add 2*pi*R  to the solution
        if ( Utils::m_2pi <= absk*(L-tmp) ) tmp += Utils::m_2pi / absk;
      }

      return tmp;

    } else {

      real_type om = atan2( b0, a0+1/k );
      if ( k < 0 ) om += Utils::m_pi;
      real_type ss = -om/k;
      real_type t  = Utils::m_2pi/abs(k);
      if      ( ss < 0 ) ss += t;
      else if ( ss > t ) ss -= t;
      return ss;
    }
  }

  // - - - - - - - - - - - - - - - - - - - - - - - - - - - - - - - - - - - - - -

  real_type
  projectPointOnCircle(
    real_type x0,
    real_type y0,
    real_type theta0,
    real_type k,
    real_type qx,
    real_type qy
  ) {
    real_type dx  = x0 - qx;
    real_type dy  = y0 - qy;
    real_type c0  = cos(theta0);
    real_type s0  = sin(theta0);
    real_type a0  = c0 * dy - s0 * dx;
    real_type b0  = s0 * dy + c0 * dx;
    real_type tmp = a0*k;

    if ( 1+2*tmp > 0 ) {
      tmp = b0/(1+tmp);
      tmp *= -Atanc(tmp*k); // lunghezza
      return tmp;
    } else {
      real_type om = atan2( b0, a0+1/k );
      if ( k < 0 ) {
        if ( om < 0 ) om += Utils::m_pi;
        else          om -= Utils::m_pi;
      }
      return -om/k;
    }
  }

  // - - - - - - - - - - - - - - - - - - - - - - - - - - - - - - - - - - - - - -

  int_type
  isPointInTriangle(
    real_type const * point,
    real_type const * p1,
    real_type const * p2,
    real_type const * p3
  ) {
    int_type d = isCounterClockwise(p1, p2, p3);
    int_type a = isCounterClockwise(p1, p2, point);
    int_type b = isCounterClockwise(p2, p3, point);
    int_type c = isCounterClockwise(p3, p1, point);
    if ( d < 0) { a = -a; b = -b; c = -c; }
    if ( a < 0 ) return -1;
    if ( b < 0 ) return -1;
    if ( c < 0 ) return -1;
    if ( a+b+c == 3 ) return 1;
    return 0;
  }

  /*\
   |  ____                  ____
   | | __ )  __ _ ___  ___ / ___|   _ _ ____   _____
   | |  _ \ / _` / __|/ _ \ |  | | | | '__\ \ / / _ \
   | | |_) | (_| \__ \  __/ |__| |_| | |   \ V /  __/
   | |____/ \__,_|___/\___|\____\__,_|_|    \_/ \___|
  \*/

  /*\
   |  _____                   _   _   _
   | |_   _|   __ _ _ __   __| | | \ | |
   |   | |    / _` | '_ \ / _` | |  \| |
   |   | |   | (_| | | | | (_| | | |\  |
   |   |_|    \__,_|_| |_|\__,_| |_| \_|
  \*/

  real_type
  BaseCurve::tx( real_type s ) const
  { return cos(theta(s)); }

  real_type
  BaseCurve::tx_D( real_type s ) const
  { return -sin(theta(s))*theta_D(s); }

  real_type
  BaseCurve::tx_DD( real_type s ) const {
    real_type th    = theta(s);
    real_type th_D  = theta_D(s);
    real_type th_DD = theta_DD(s);
    real_type C     = cos(th);
    real_type S     = sin(th);
    return -(th_DD*S+(th_D*th_D)*C);
  }

  real_type
  BaseCurve::tx_DDD( real_type s ) const {
    real_type th     = theta(s);
    real_type th_D   = theta_D(s);
    real_type th_DD  = theta_DD(s);
    real_type th_DDD = theta_DDD(s);
    real_type C      = cos(th);
    real_type S      = sin(th);
    return (th_D*th_D*th_D-th_DDD)*S-3*th_DD*th_D*C;
  }

  // . . . . . . . . . . . . . . . . . . . . . . . . . . . . . . . . . . . . .

  real_type
  BaseCurve::ty( real_type s ) const
  { return sin(theta(s)); }

  real_type
  BaseCurve::ty_D( real_type s ) const
  { return cos(theta(s))*theta_D(s); }

  real_type
  BaseCurve::ty_DD( real_type s ) const {
    real_type th    = theta(s);
    real_type th_D  = theta_D(s);
    real_type th_DD = theta_DD(s);
    real_type C     = cos(th);
    real_type S     = sin(th);
    return th_DD*C-(th_D*th_D)*S;
  }

  real_type
  BaseCurve::ty_DDD( real_type s ) const {
    real_type th     = theta(s);
    real_type th_D   = theta_D(s);
    real_type th_DD  = theta_DD(s);
    real_type th_DDD = theta_DDD(s);
    real_type C      = cos(th);
    real_type S      = sin(th);
    return (th_DDD-th_D*th_D*th_D)*C-3*(th_DD*th_D)*S;
  }

  /*\
   |         __  __          _
   |   ___  / _|/ _|___  ___| |_
   |  / _ \| |_| |_/ __|/ _ \ __|
   | | (_) |  _|  _\__ \  __/ |_
   |  \___/|_| |_| |___/\___|\__|
  \*/

  real_type
  BaseCurve::X_ISO( real_type s, real_type offs ) const
  { return X(s) + offs * nx_ISO(s); }

  real_type
  BaseCurve::Y_ISO( real_type s, real_type offs ) const
  { return Y(s) + offs * ny_ISO(s); }

  real_type
  BaseCurve::X_ISO_D( real_type s, real_type offs ) const
  { return X_D(s) + offs * nx_ISO_D(s); }

  real_type
  BaseCurve::Y_ISO_D( real_type s, real_type offs ) const
  { return Y_D(s) + offs * ny_ISO_D(s); }

  real_type
  BaseCurve::X_ISO_DD( real_type s, real_type offs ) const
  { return X_DD(s) + offs * nx_ISO_DD(s); }

  real_type
  BaseCurve::Y_ISO_DD( real_type s, real_type offs ) const
  { return Y_DD(s) + offs * ny_ISO_DD(s); }

  real_type
  BaseCurve::X_ISO_DDD( real_type s, real_type offs ) const
  { return X_DDD(s) + offs * nx_ISO_DDD(s); }

  real_type
  BaseCurve::Y_ISO_DDD( real_type s, real_type offs ) const
  { return Y_DDD(s) + offs * ny_ISO_DDD(s); }

  // . . . . . . . . . . . . . . . . . . . . . . . . . . . . . . . . . . . . .

  void
  BaseCurve::eval_ISO(
    real_type   s,
    real_type   offs,
    real_type & x,
    real_type & y
  ) const {
    real_type nx, ny;
    nor_ISO( s, nx, ny );
    eval( s, x, y );
    x += offs * nx;
    y += offs * ny;
  }

  void
  BaseCurve::eval_ISO_D(
    real_type   s,
    real_type   offs,
    real_type & x_D,
    real_type & y_D
  ) const {
    real_type nx_D, ny_D;
    nor_ISO_D( s, nx_D, ny_D );
    eval_D( s, x_D, y_D );
    x_D += offs * nx_D;
    y_D += offs * ny_D;
  }

  void
  BaseCurve::eval_ISO_DD(
    real_type   s,
    real_type   offs,
    real_type & x_DD,
    real_type & y_DD
  ) const {
    real_type nx_DD, ny_DD;
    nor_ISO_D( s, nx_DD, ny_DD );
    eval_DD( s, x_DD, y_DD );
    x_DD += offs * nx_DD;
    y_DD += offs * ny_DD;
  }

  void
  BaseCurve::eval_ISO_DDD(
    real_type   s,
    real_type   offs,
    real_type & x_DDD,
    real_type & y_DDD
  ) const {
    real_type nx_DDD, ny_DDD;
    nor_ISO_D( s, nx_DDD, ny_DDD );
    eval_DDD( s, x_DDD, y_DDD );
    x_DDD += offs * nx_DDD;
    y_DDD += offs * ny_DDD;
  }

  // - - - - - - - - - - - - - - - - - - - - - - - - - - - - - - - - - - - - - -

}

// EOF: G2lib.cc
```

### Quick search

### Table of Contents

- Matlab Interface Manual
- C++ API
- MATLAB API

«
hide menu

menu
sidebar
»

### Navigation

- index
- toc
- Clothoids »
- Program Listing for File G2lib.cc

© Copyright 2021, Enrico Bertolazzi and Marco Frego.
Created using Sphinx 4.2.0.
